# Supplementary material for: Characterization of spray-dried Gac aril extract and estimated shelf life of β-carotene and lycopene
Source: PeerJ. 2021 Mar 24;9:e11134. doi: 10.7717/peerj.11134 (PMC8000464; doi:10.7717/peerj.11134)
Supplement: File S1 [file peerj-09-11134-s001.docx]

Table β-carotene and lycopene content of sprayed dried Gac aril powders of different storage time and temperature

| Day | Storage temperature (°C) | | | | | |
| --- | --- | --- | --- | --- | --- | --- |
|  | 30 | | 45 | | 55 | |
|  | β-carotene (mg) | Lycopene (mg) | β-carotene (mg) | Lycopene (mg) | β-carotene (mg) | Lycopene (mg) |
| 0 | 1.403±0.019 | 0.225±0.004 | 1.403±0.019 | 0.225±0.004 | 1.403±0.019 | 0.225±0.004 |
| 3 | 1.357±0.047 | 0.176±0.010 | 0.980±0.034 | 0.128±0.001 | 0.465±0.027 | 0.070±0.002 |
| 6 | 1.301±0.021 | 0.113±0.003 | 0.480±0.011 | 0.117±0.003 | 0.285±0.021 | 0.066±0.001 |
| 9 | 1.297±0.049 | 0.101±0.008 | 0.451±0.019 | 0.116±0.001 | 0.244±0.016 | 0.062±0.001 |
| 12 | 1.223±0.033 | 0.099±0.002 | 0.412±0.022 | 0.105±0.003 | 0.188±0.015 | 0.051±0.001 |
| 15 | 1.149±0.060 | 0.098±0.004 | 0.395±0.017 | 0.102±0.005 | 0.158±0.003 | 0.046±0.002 |
| 18 | 0.903±0.018 | 0.092±0.006 | 0.401±0.031 | 0.100±0.002 | 0.154±0.004 | 0.020±0.002 |
| 21 | 0.887±0.023 | 0.068±0.004 | 0.229±0.013 | 0.077±0.001 | 0.141±0.010 | nd |
| 24 | 0.798±0.063 | 0.033±0.004 | 0.233±0.009 | nd | nd | nd |
| 27 | 0.494±0.031 | nd | 0.178±0.011 | nd | nd | nd |
| 30 | 0.437±0.040 | nd | 0.150±0.010 | nd | nd | nd |
| 33 | 0.374±0.042 | nd | 0.128±0.017 | nd | nd | nd |
| 36 | 0.241±0.012 | nd | nd | nd | nd | nd |
| 39 | 0.213±0.006 | nd | nd | nd | nd | nd |
| 42 | 0.117±0.014 | nd | nd | nd | nd | nd |
| 45 | nd | nd | nd | nd | nd | nd |

Nd = not detected.
