# Supplementary material for: Characterization of spray-dried Gac aril extract and estimated shelf life of β-carotene and lycopene
Source: PeerJ. 2021 Mar 24;9:e11134. doi: 10.7717/peerj.11134 (PMC8000464; doi:10.7717/peerj.11134)
Supplement: File S2 [file peerj-09-11134-s002.pdf]

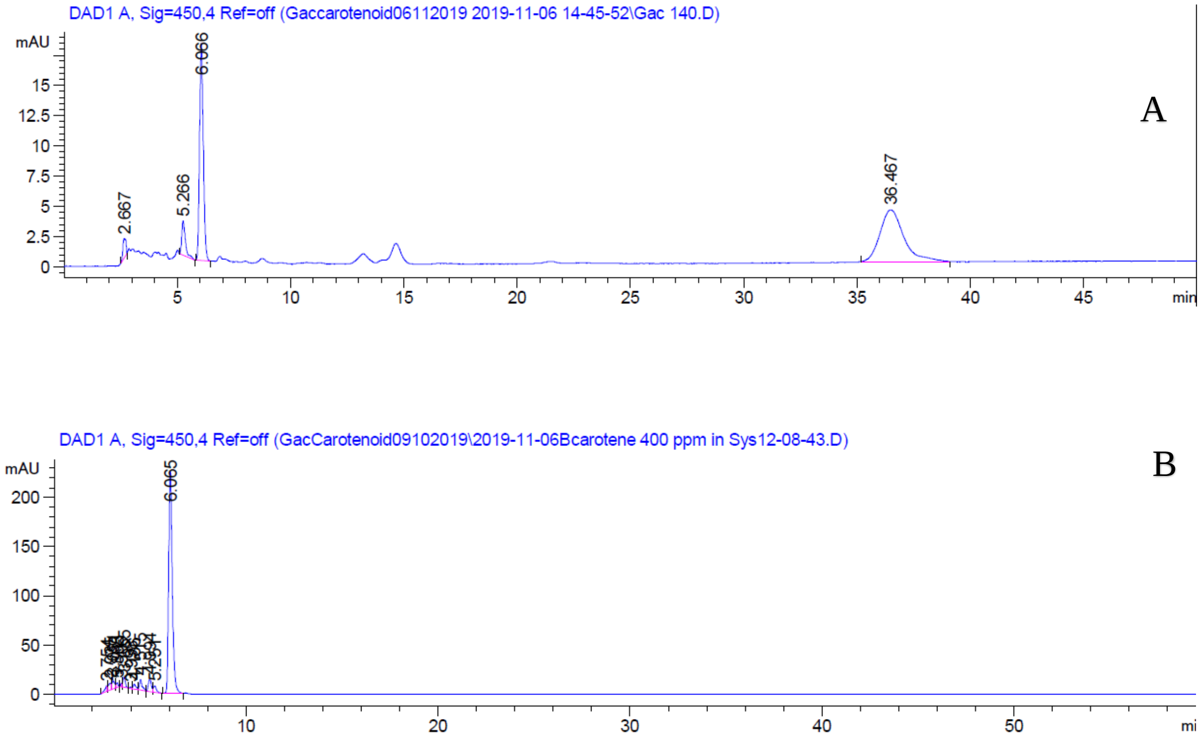

β-carotene (A) and lycopene (B) standard at 450 nm.

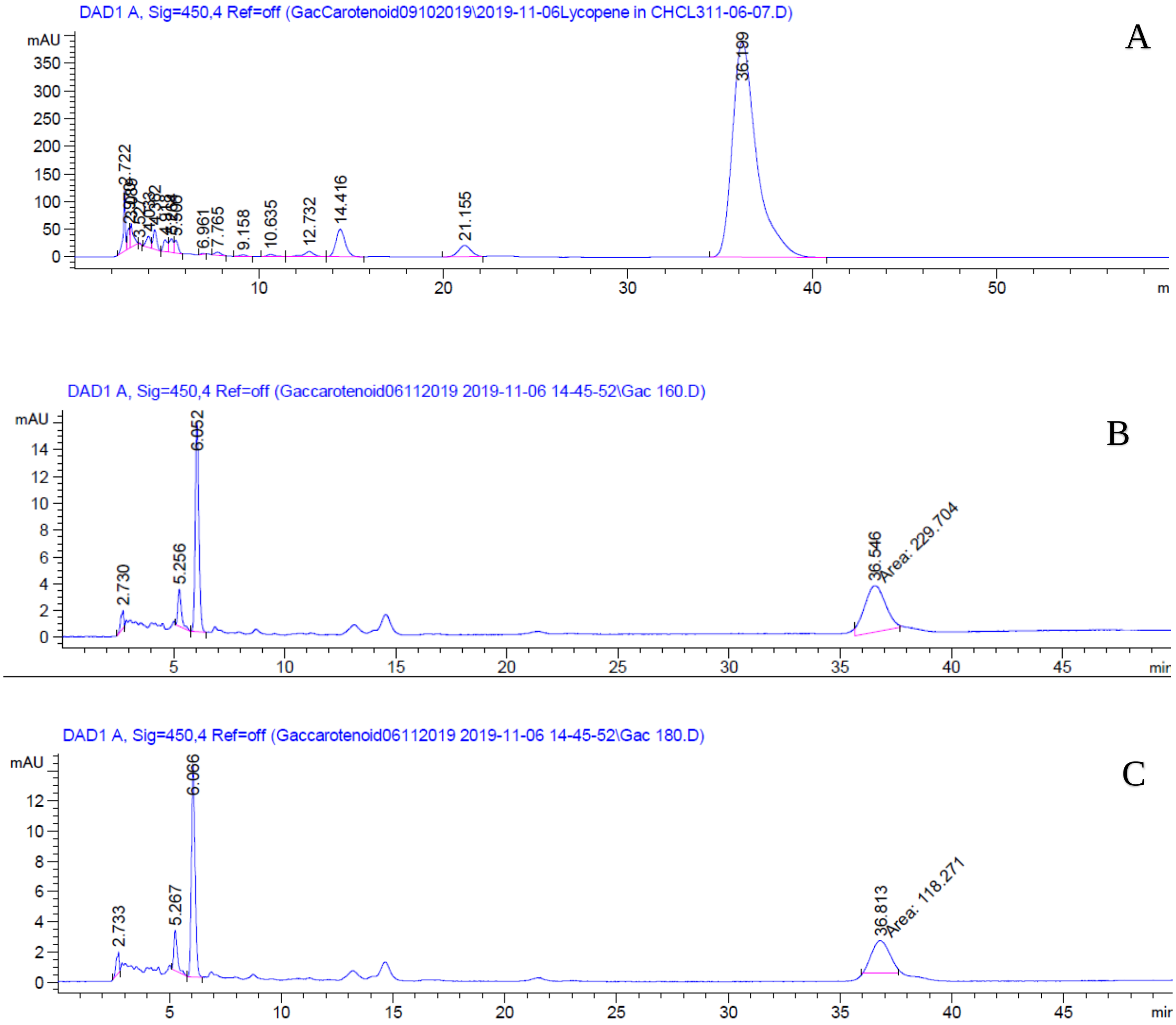

β-carotene (A) and lycopene (B) of Gac aril powders dried at 140°C (A), 160°C (B), 180°C (C) determine at 450 nm
